# CFTR interacts with Hsp90 and regulates the phosphorylation of AKT and ERK1/2 in colorectal cancer cells

**DOI:** 10.1002/2211-5463.12641

**Published:** 2019-04-29

**Authors:** Kaisheng Liu, Hongtao Jin, Yaomin Guo, Ying Liu, Yong Wan, Pan Zhao, Zhifan Zhou, Jianhong Wang, Maolin Wang, Chang Zou, Weiqing Wu, Zhiqiang Cheng, Yong Dai

**Affiliations:** ^1^ The First Affiliated Hospital of Southern University of Science and Technology The Second Clinical Medical College of Jinan University Shenzhen People's Hospital Shenzhen China; ^2^ Faculty of Medicine School of Biomedical Sciences The Chinese University of Hong Kong Hong Kong SAR China; ^3^ School of Medicine Health Science Centre Shenzhen University Shenzhen China

**Keywords:** AKT, cystic fibrosis transmembrane conductance regulator, extracellular signal‐regulated kinase ½, heat‐shock protein 90, mitochondria

## Abstract

Cystic fibrosis (CF) is caused by mutations in the cystic fibrosis transmembrane conductance regulator (*CFTR*) gene. CF cells and tissues exhibit various mitochondrial abnormalities. However, the underlying molecular mechanisms remain elusive. Here, we examined the mechanisms through which CFTR regulates Bcl‐2 family proteins, which in turn regulate permeabilization of the mitochondrial outer membrane. Notably, inhibition of CFTR activated Bax and Bad, but inhibited Bcl‐2. Moreover, degradation of phosphorylated extracellular signal‐regulated kinase 1/2 (ERK1/2) and AKT increased significantly in CFTR‐knockdown cells. Dysfunction of CFTR decreased heat‐shock protein 90 (*Hsp90*) mRNA levels, and CFTR was found to interact with Hsp90. Inhibition of Hsp90 by SNX‐2112 induced the degradation of phosphorylated AKT and ERK1/2 in Caco2 and HRT18 cells. These findings may help provide insights into the physiological role of CFTR in CF‐related diseases.

**Abbreviations**
CF: cystic fibrosis
CFTR: cystic fibrosis transmembrane conductance regulator
ERK1/2: extracellular signal‐regulated kinase 1/2
Hsp90: heat‐shock protein 90
shRNA: short hairpin RNA
ΔF508: deletion of the phenylalanine at position 508

Cystic fibrosis (CF) is an autosomal recessive genetic disease that is most common in Caucasians that is caused by mutations in the cystic fibrosis transmembrane conductance regulator (*CFTR*) gene 1. *CFTR* encodes a cAMP‐regulated anion channel expressed at the apical membrane of epithelial cells in various organs 2. The CFTR channel can transport chloride, bicarbonate, and glutathione 3, 4. CFTR interacts either directly or indirectly with some proteins by forming a macromolecular complex 5, 6, 7, 8. More than 2000 mutations have been identified in CF, and deletion of the phenylalanine at position 508 (ΔF508) is the most frequent mutation occurring in over 80% of patients with CF 9, 10, 11, 12. *CFTR* mutations affect the mucosal physiology of the respiratory, digestive, and reproductive tracts, leading to systemic diseases, including pancreatic insufficiency, focal biliary cirrhosis, infertility, intestinal obstruction, increased cancer risk, and chronic airway obstruction 13, 14.

Interestingly, mitochondrial abnormalities have been found in CF cells and tissues 15, 16, 17. For example, calcium uptake and oxygen consumption are altered in the mitochondria of patients with CF 18. Additionally, fragmentation of mitochondria and a decrease in the mitochondrial membrane potential were observed in CF cells compared with control cells 19. Mitochondrial proteins have also been shown to exhibit differences in the two‐dimensional electrophoretic patterns in CF 20. Moreover, reactive oxygen species and autophagy may be related to mitochondrial dysfunction in patients with CF 21, 22, 23. However, the underlying molecular mechanisms remain elusive.

Thus, in this study, we aimed to evaluate the possible roles of CFTR in mitochondrial abnormalities. Our results provided important insights into the roles of CFTR in mitochondrial dysfunction via a mechanism involving heat‐shock protein 90 (Hsp90)‐mediated extracellular signal‐regulated kinase (ERK) 1/2 and AKT, suggesting the involvement of CFTR in CF‐related diseases.

## Materials and methods

### Antibodies and reagents

Anti‐C‐terminal‐CFTR (CFTR‐C) antibodies were purchased from Alomone Labs (Jerusalem, Israel) and anti‐CFTR antibodies were from Cell Signaling Technology (Danvers, MA, USA). Anti‐β‐actin antibodies were obtained from Santa Cruz Biotechnology (Santa Cruz, CA, USA). Anti‐Bcl‐2, anti‐Bax, anti‐Bad, anti‐ERK1/2, anti‐AKT, anti‐phospho‐ERK1/2, and anti‐phospho‐Akt antibodies were obtained from Cell Signaling Technology.

### Cell culture and lentiviral transduction

The human colon cancer cell lines Caco2 and HRT18 (American Type Culture Collection, Manassas, VA, USA) were cultured in MEM or RPMI‐1640, respectively, supplemented with 10% fetal bovine serum and 1% penicillin–streptomycin in an incubator at 37 °C in an atmosphere containing 5% CO_2_.

Lentiviral transduction particles encoding short hairpin RNA (shRNA) against CFTR were purchased from Jima Inc. (Shanghai, China). The sequences (5′ to 3′) of the two shRNAs were GAAGTAGTGATGGAGAATGTA and TTGGAAAGGAGACTAACAAGT, respectively. These two shRNA duplexes were from two different regions of human *CFTR* mRNA. Cells were plated in 24‐well cell culture plates, incubated with 5 μL lentivirus (1 × 10^9^ TU·mL^−1^, 1 × 10^9^ TU·mL^−1^) for 24 or 48 h, and collected for analysis. Viral vectors containing noncoding shRNA were used as a control.

### Western blotting

Cells were lysed in RIPA buffer with 1 : 100 protein inhibitor for 30 min on ice. The supernatants were collected as total protein after centrifugation at 15 000 ***g*** for 30 min. Equal amounts of protein were separated by sodium dodecyl sulfate polyacrylamide electrophoresis followed by western blotting. The protein bands were visualized by enhanced chemiluminescence (Millipore, Billerica, MA, USA) according to the manufacturer's instructions, and data were quantified using densitometry analysis. Experiments were repeated three times, and the bands were scanned and quantified.

### Immunohistochemistry and fluorescent immunocytochemistry

The sections were incubated with phosphate‐buffered saline (PBS) for 5–10 min and retrieval buffer (citrate buffer, pH 6.0) for 30 min. After washing in PBS, the sections were incubated with primary antibodies overnight at 4 °C. The sections were then incubated with secondary antibodies for 60 min at room temperature. Finally, the sections were mounted with coverslips and visualized by microscopy.

### RNA extraction and real‐time quantitative polymerase chain reaction (RT/qPCR)

Total RNA was extracted using an RNeasy Mini Kit (Qiagen GmbH, Hilden, Germany) and was reverse‐transcribed using a PrimeScript RT Master Mix kit (TaKaRa, Dalian, China) for standard real‐time PCR analysis. RT/qPCR was performed using a TB Green Premix EX Taq II detection system in a Roche LightCycler 96 qPCR machine (Roche Diagnostics, Mannheim, Germany). Glyceraldehyde 3‐phosphate dehydrogenase was used as a control. The gene‐specific primers are summarized in Table S1.

### Co‐immunoprecipitation (Co‐IP)

CFTR was overexpressed in 293T cells, and cells were cultured for 48 h. Cell lysates were collected in IP Lysis/Wash Buffer with a Pierce Crosslink Magnetic IP/Co‐IP Kit (cat. no.: 88805; Thermo Scientific, Rockford, IL, USA). Cell lysates were then incubated with Protein A/G Magnetic Beads bound to CFTR antibodies for 2 h at room temperature. The beads were washed with IP wash buffer and then eluted in elution buffer to collect the target protein. Co‐IP was performed according to the instructions provided by the kit manufacturer.

### Three‐dimensional (3D) structure of the CFTR/Hsp90 complex

Three‐dimensional structures of CFTR (5TF7) and Hsp90 (6GPR) were downloaded from RCSB PDB. The complex was built using pymol, version 2.0. The model contained the amino acids residues predicted as possible interacting sites 24, 25.

### Statistical analysis

Data were expressed as means ± standard deviations. Differences in measured variables between two groups were analyzed using Student's *t*‐tests, and differences between more than two groups were analyzed by one‐way analysis of variance. Results with *P* values of < 0.05 were considered statistically significant.

## Results

### Knockdown of CFTR

**Figure 1:**
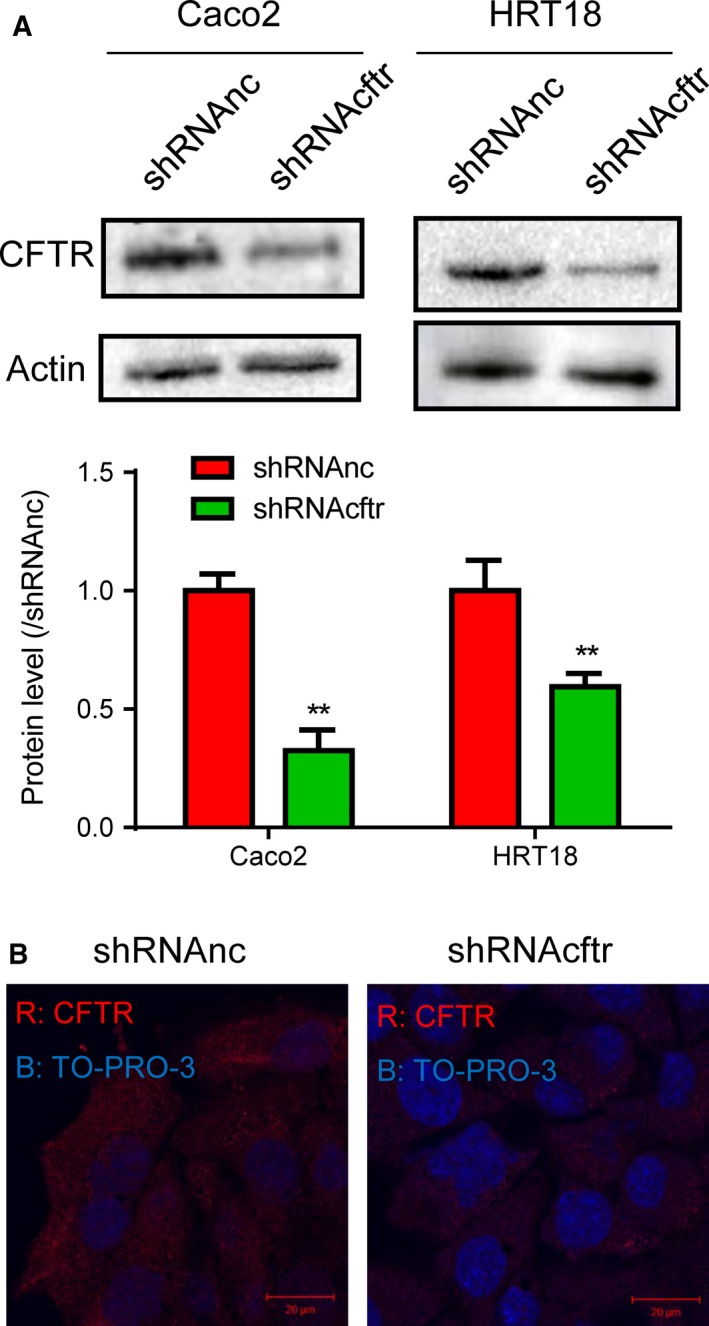
Knockdown of CFTR. (A) Western blot analysis of the effects of shRNA on CFTR expression in Caco2 and HRT18 cells. Actin was used as a loading control. Quantification of western blotting results is shown in the bottom panel. Data were expressed as means ± standard deviations. Differences in measured variables between two groups were analyzed using Student's *t*‐tests. **P < 0.01 (*n* = 3). (B) Immunofluorescence staining of CFTR in Caco2^shRNAnc^ and Cacao2^shRNAcftr^ cells. Scale bar = 20 μm.

Initially, we assessed the effects of shRNA against CFTR by western blotting. Caco2 and HRT18 cells were transfected with lentivirus for 48 h. Western blot analysis of CFTR protein was then performed. The results showed that CFTR was significantly downregulated (Fig. 1A). Moreover, in Caco2 cells, CFTR was not expressed at the apical membrane after knockdown (Fig. 1B).

### Knockdown of CFTR induced mitochondrial dysfunction by regulating the phosphorylation of AKT and ERK1/2

**Figure 2:**
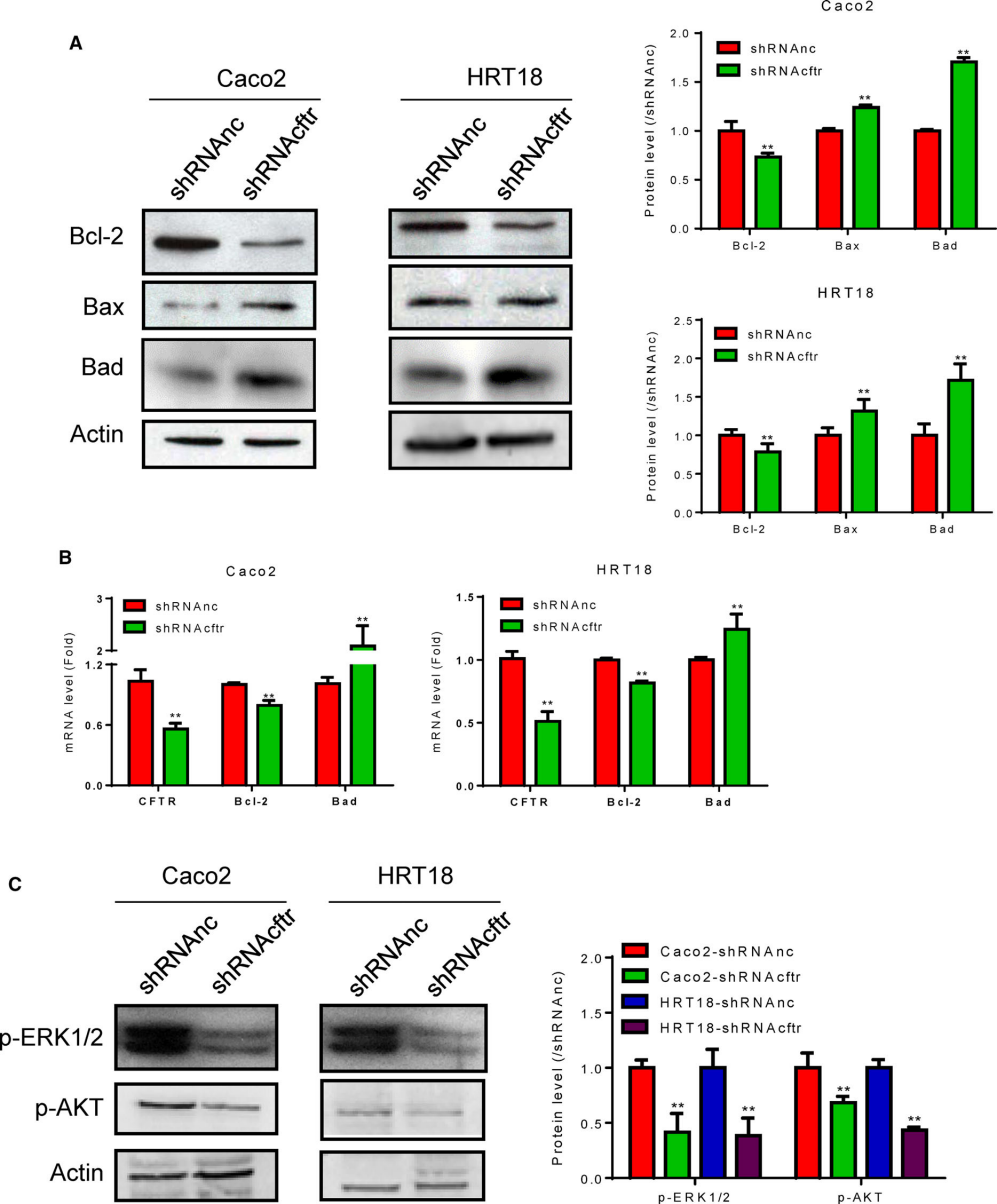
Knockdown of CFTR induced mitochondrial dysfunction and degradation of phospho‐AKT and phospho‐ERK1/2. Caco2 cells and HRT18 cells were transfected with lentivirus, cultured, and collected. (A) Equal amounts of whole cell lysates (25–50 μg) were subjected to western blotting. The expression of Bcl‐2 family proteins was detected. Actin was used as a loading control. Quantification of western blot results is shown in the right panel. (B) mRNA levels of *CFTR*,* Bcl‐2*, and *Bad* were detected by RT/qPCR in CFTR‐knockdown Caco2 and HRT18 cells. (C) Western blot analysis of phospho‐AKT and phospho‐ERK1/2 in CFTR‐knockdown Caco2 and HRT18 cells. Actin was used as a loading control. Quantification of western blotting results is shown in the right panel. All data were expressed as means ± standard deviations. Differences in measured variables between two groups were analyzed using Student's *t*‐tests. **P < 0.01 (*n* = 3).

To determine whether CFTR knockdown affected mitochondrial function in Caco2 and HRT18 cells, we performed western blot analysis of Bcl‐2 family members, including Bcl‐2, Bad, and Bax. The results showed that Bax and Bad were upregulated, and Bcl‐2 was downregulated in CFTR‐knockdown cells (Fig. 2A). In addition, the mRNA levels of *Bax* and *Bad* were upregulated, whereas *Bcl‐2* mRNA was downregulated in CFTR‐knockdown cells (Fig. 2B).

To further explore the molecular mechanisms involved in mitochondrial dysfunction induced by CFTR knockdown, we measured the phosphorylation of AKT and ERK1/2, which regulate Bcl‐2 family proteins. Western blot analysis indicated that phospho‐AKT and phospho‐ERK1/2 levels were decreased in CFTR‐knockdown cells (Fig. 2C). These findings suggested that knockdown of CFTR induced mitochondrial dysfunction in Caco2 and HRT18 cells by regulating the phosphorylation of AKT and ERK1/2.

### CFTR interacted with Hsp90

**Figure 3:**
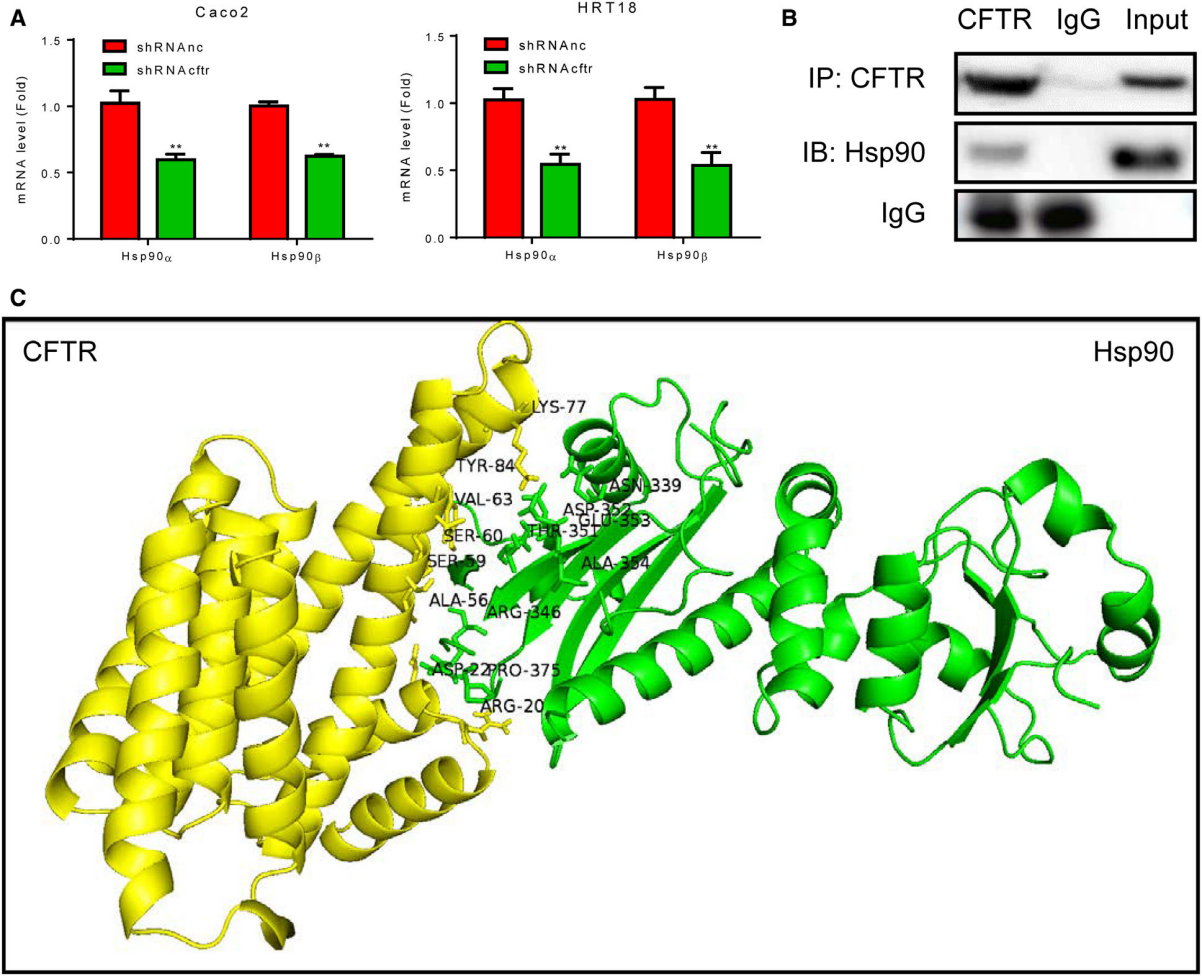
The relationship between CFTR and Hsp90. Caco2 cells and HRT18 cells were transfected with lentivirus, cultured, and collected. (A) mRNA levels of *Hsp90* were detected by RT/qPCR in CFTR‐knockdown Caco2 and HRT18 cells. Data were expressed as means ± standard deviations. Differences in measured variables between two groups were analyzed using Student's *t*‐tests. **P < 0.01 (*n* = 3). (B) Co‐IP study in 293T cells with CFTR overexpression. Hsp90 immunoprecipitated with anti‐CFTR antibodies. The blots shown are representative images from three independent experiments. (C) Model of the CFTR/Hsp90 complex. The amino acid residues identified as possible interacting sites were labeled using their common three‐letter abbreviations.

AKT and ERK1/2 are regulated by Hsp90 26; indeed, inhibition of Hsp90 induces the degradation of phospho‐AKT and phospho‐ERK1/2 27, 28, 29. Therefore, we next investigated the protein levels of Hsp90 in CFTR‐knockdown cells. The results showed that both Hsp90α and Hsp90β decreased significantly in CFTR‐knockdown Caco2 and HRT18 cells (Fig. 3A). To further investigate the relationships between CFTR and Hsp90, we performed Co‐IP in CFTR‐overexpressing 293T cells. The results showed that Hsp90 could be pulled down by CFTR (Fig. 3B). Although the 3D structures of both CFTR and Hsp90 were solved, the structure of the complex between these two proteins has not been determined experimentally yet. Therefore, we then built a model of the protein–protein interaction with PyMOL to predict the complex (Fig. 3C). These results indicated that degradation of phospho‐ERK1/2 and phospho‐AKT in CFTR‐knockdown cells might be associated with decreased Hsp90 expression.

### Phospho‐ERK1/2 and phospho‐AKT were regulated by Hsp90

**Figure 4:**
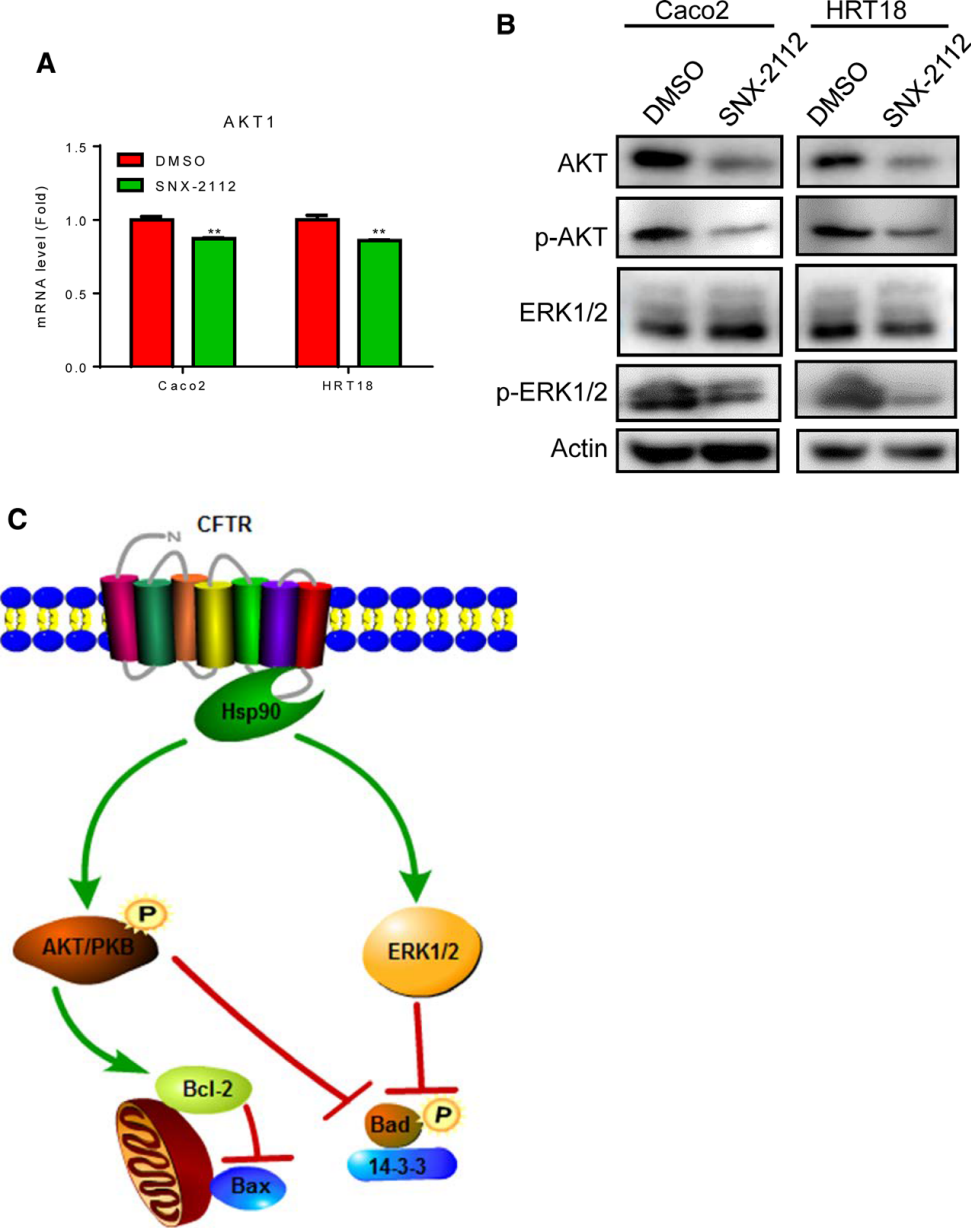
Suppression of ERK1/2 and AKT phosphorylation by Hsp90 inhibition. Caco2 and HRT18 cells were treated with 0.25 μm SNX‐2112 for 48 h and then collected. (A) mRNA levels of *Akt1* were detected by RT/qPCR in SNX‐2112‐treated Caco2 and HRT18 cells. Data were expressed as means ± standard deviations. Differences in measured variables between two groups were analyzed using Student's *t*‐tests. **P < 0.01 (*n* = 3). (B) Equal amounts of whole cell lysates (25–50 μg) were subjected to western blotting. Levels of total and phosphorylated AKT and ERK1/2 proteins were detected. Actin was used as a loading control. (C) Schematic illustration of the molecular mechanisms through which disruption of the CFTR/Hsp90 interaction induced mitochondrial dysfunction.

To demonstrate that degradation of phospho‐ERK1/2 and phospho‐AKT was regulated by Hsp90, Caco2 and HRT18 cells were treated with 0.25 μM SNX‐2112, a selective Hsp90 inhibitor, for 48 h 30. The results showed that *Akt* mRNA levels were downregulated (Fig. 4A), and the levels of AKT protein, phospho‐AKT, and phospho‐ERK1/2 were also decreased (Fig. 4B) in SNX‐2112‐treated cells. Taken together, these data suggest that CFTR knockdown may induce mitochondrial dysfunction via Hsp90‐mediated degradation of phospho‐ERK1/2 and phospho‐AKT (Fig. 4C).

## Discussion

CFTR acts as a chloride channel and can interact directly or indirectly with several proteins by forming a complex to regulate cell signaling processes 31. Knockdown of CFTR suppresses the proliferation of ovarian cancer *in vitro* and *in vivo* 32. In contrast, CFTR has also been reported to function as a tumor suppressor 33, and downregulation or mutation of CFTR activates proliferation, invasion, migration, and the epithelial–mesenchymal transition in breast cancer and prostate cancer 34, 35, 36. Hyperproliferation has been observed in CF airways 37, and downregulation of CFTR has been reported in cancer tissues 33. In addition, mitochondrial abnormalities have been reported in CF cells or tissues 38. Differential expression of genes or proteins and alterations in calcium homeostasis, membrane potential, reactive oxygen species, and complex I activity have all been reported in mitochondria in the context of CFTR knockdown 39. These mitochondrial effects in turn induce changes in the ratio of reduced/oxidized glutathione and trigger proliferation or apoptotic events. Moreover, these alterations may influence the phenotype or clinical manifestations of CF 19, 21, 38, 40. However, the molecular mechanisms underlying mitochondrial dysfunction remain elusive.

In this study, we showed, for the first time, that disruption of the CFTR/Hsp90 interaction induced mitochondrial dysfunction via degradation of phospho‐AKT and phospho‐ERK1/2 in Caco2 and HRT18 cells. In CFTR‐knockdown cells, Bax and Bad were activated, whereas Bcl‐2 was inhibited, indicating the involvement of mitochondrial dysfunction. The mitochondria play important roles in apoptosis and proliferation 40. Thus, further studies are needed to evaluate the roles of CFTR in mitochondrial dysfunction and subsequent cell proliferation and apoptosis.

AKT and ERK1/2 are crucial proteins that regulate the Bcl‐2 family 41. AKT upregulates Bcl‐2 expression through cAMP‐response element‐binding protein 42. Bad is a distant member of the Bcl‐2 family and can be phosphorylated by AKT and ERK1/2 43, 44, 45. In this study, both phospho‐ERK1/2 and phospho‐AKT levels were significantly decreased in CFTR‐knockdown cells, suggesting that mitochondrial dysfunction induced by CFTR dysfunction may be regulated by the AKT and ERK1/2 pathways. AKT and ERK1/2 are the target proteins of Hsp90 46, and inhibition of Hsp90 induces degradation of these proteins 47. In this study, we found that Hsp90 was downregulated in CFTR‐knockdown cells. Hsp90 interacted with CFTR. Hsp90 inhibitors induce the degradation of AKT and ERK1/2. Additional studies are needed to clarify the mechanisms mediating the interactions between CFTR and Hsp90. The regulation mechanisms of CFTR and Hsp90 on mitochondria, especially how CFTR and Hsp90 work together to maintain the function of mitochondria, will be investigated in the future work.

Taken together, our findings revealed a previously unidentified role of CFTR defects in mitochondrial abnormalities via the Hsp90‐mediated degradation of phosphorylated ERK1/2 and AKT. These results provided novel insights into the physiological functions of CFTR and CF‐related diseases.

## Conflict of interest

The authors declare no conflict of interest.

## Author contributions

KL conceived and designed the experiments. KL, YG, and YL performed the experiments and analyzed the data. KL, HJ, YW, PZ, ZZ, JW, MW, CZ, WW, ZC, and YD contributed reagents/materials/analysis tools. KL wrote the manuscript.

## Supporting information


**Table S1.** The oligonucleotide primer sequences for RT‐qPCR.
