# Supplementary material for: CFTR interacts with Hsp90 and regulates the phosphorylation of AKT and ERK1/2 in colorectal cancer cells
Source: FEBS Open Bio. 2019 Apr 29;9(6):1119–27. doi: 10.1002/2211-5463.12641 (PMC6551490; doi:10.1002/2211-5463.12641)
Supplement: Supplementary file 1 — Table S1. The oligonucleotide primer sequences for RT‐qPCR. [file FEB4-9-1119-s001.docx]

Supplementary Table S1. The oligonucleotide primer sequences for RT-qPCR.

| Gene |  | Primer Sequence |
| --- | --- | --- |
| Human Hsp90α | Up | AGGAGGTTGAGACGTTCGC |
|  | Down | AGAGTTCGATCTTGTTTGTTCGG |
| Human Hsp90β | Up | AGAAATTGCCCAACTCATGTCC |
|  | Down | ATCAACTCCCGAAGGAAAATCTC |
| Human GAPDH | Up | GGAGCGAGATCCCTCCAAAAT |
|  | Down | GGCTGTTGTCATACTTCTCATGG |
| Human Akt1 | Up | AGCGACGTGGCTATTGTGAAG |
|  | Down | GCCATCATTCTTGAGGAGGAAGT |
| Human CFTR | Up | GTGTGATTCCACCTTCTCCAA |
|  | Down | GCCTGGCACCATTAAAGAAA |
| Human Bcl-2 | Up | GGTGGGGTCATGTGTGTGG |
|  | Down | CGGTTCAGGTACTCAGTCATCC |
| Human Bad | Up | CCCAGAGTTTGAGCCGAGTG |
|  | Down | CCCATCCCTTCGTCGTCCT |

Up: forward primer; Down: reverse primer.
